# Bile duct stone removal via an endoscopic ultrasound guided-hepaticogastrostomy route created using a B1 puncture technique

**DOI:** 10.1055/a-2919-0092

**Published:** 2026-08-27

**Authors:** Fumihiko Asahina, Ryota Sagami, Takeshi Agario, Yasuhisa Hiroshima, Kazuhiro Mizukami

**Affiliations:** 1 Department of Gastroenterology 13235 Oita University Faculty Of MedicineYufuOita PrefectureJapan; 2 Department of Advanced Gastrointestinal Cancer Medicine 13235 Oita University Faculty Of MedicineYufuOita PrefectureJapan

An 83-year-old woman was admitted for the endoscopic management of recurrent cholangitis localized to segment 1, caused by a large stone (≥ 15 mm) in the proximal B1 bile duct ( Fig. 1a ). She had previously undergone endoscopic ultrasound–guided hepaticogastrostomy (EUS-HGS) for the treatment of bile duct stones in segments B2 ( Fig. 1b ). Balloon enteroscopy–assisted endoscopic retrograde cholangiopancreatography (BE-ERCP) failed because the guidewire could not be advanced into the B1 branch ( Video 1 ).

**Fig. 1:**
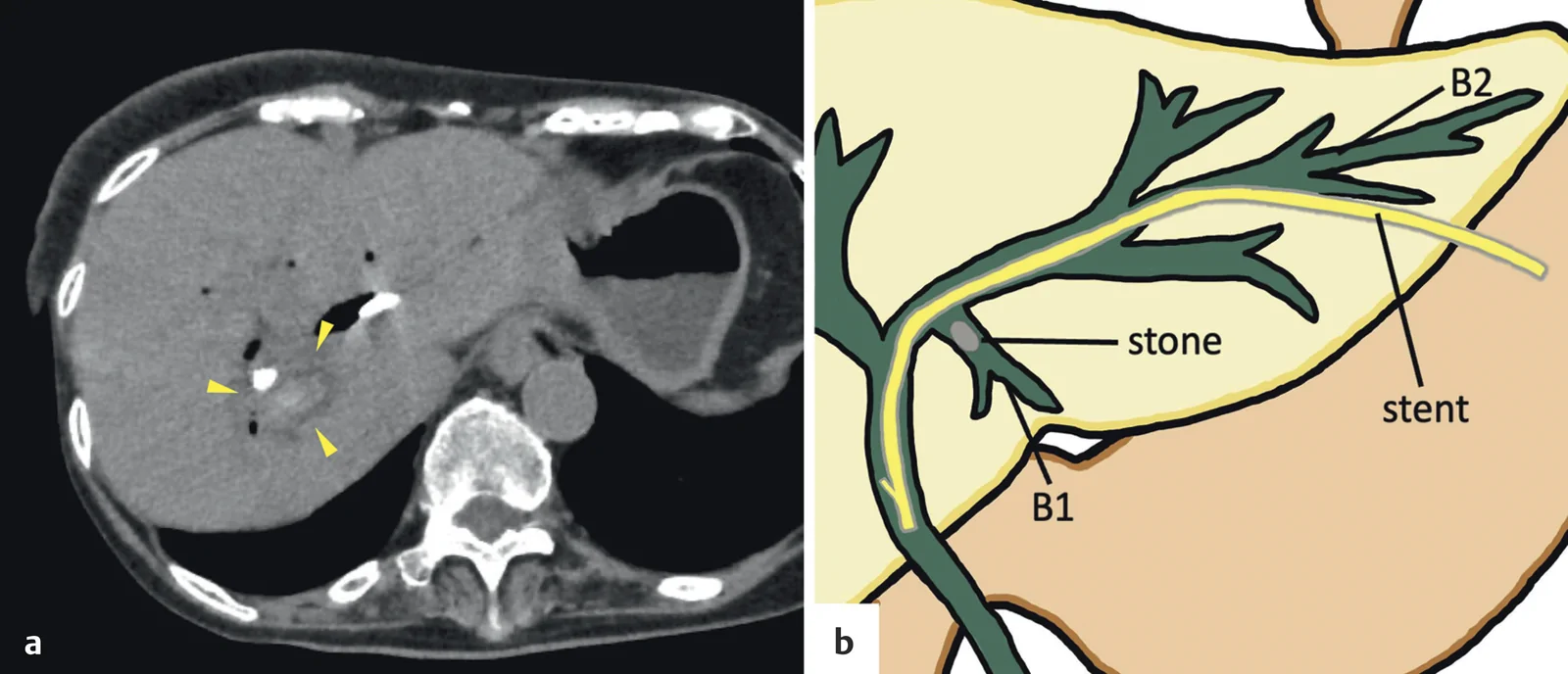
Images show the B1 bile duct stone and the EUS-HGS route via the B2 bile duct. ( **a** ) Computed tomography showing a large stone in the proximal B1 bile duct (yellow arrowheads). ( **b** ) Schematic illustration of the B1 route with a downstream stone and the B2 route with the placement of an endoscopic ultrasound–guided hepaticogastrostomy (EUS-HGS) stent.

**Video 1** Endoscopic ultrasound–guided hepaticogastrostomy via the B1 bile duct. Despite the technical difficulty of B1 branch puncture, peroral cholangioscopy–guided electrohydraulic lithotripsy enabled the complete removal of a large intrahepatic bile duct stone in the B1 segment.


Upon endoscopic ultrasound, the B1 branch was successfully punctured using a 22-gauge needle ( Fig. 2a ). A 0.018-inch guidewire was advanced across the stone into the common bile duct. Subsequently, a double-lumen cannula was used, with a second guidewire providing stable access for stent placement. A dedicated plastic stent (7 Fr×14 cm) for EUS-HGS was successfully deployed ( Fig. 2b ).

**Fig. 2:**
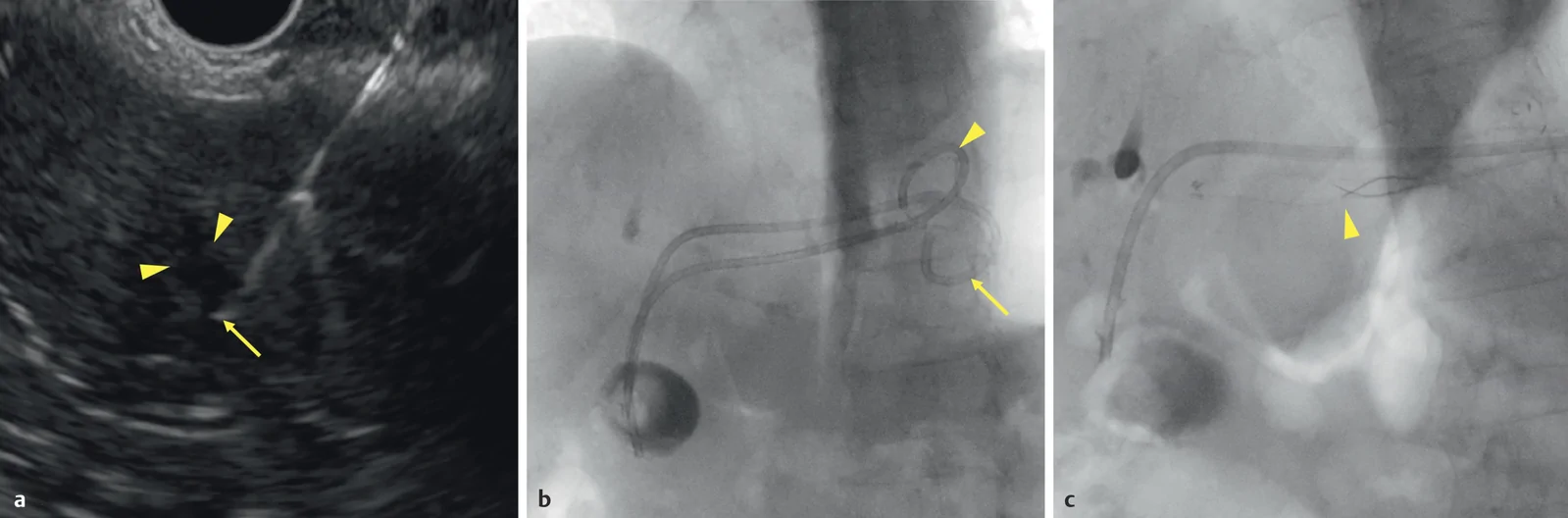
Images obtained during endoscopic ultrasound–guided hepaticogastrostomy. ( **a** ) An endoscopic ultrasound image showing puncture of the B1 bile duct (yellow arrowheads) with a needle (yellow arrow). ( **b** ) A newly deployed plastic stent in the B1 bile duct (yellow arrowhead) adjacent to a previously deployed stent in the B3 bile duct (yellow arrow). ( **c** ) Additional deployment of a self-expandable metal stent (yellow arrowhead).

Several weeks later, peroral cholangioscopy (POCS) could not be advanced because the EUS-HGS tract remained insufficiently dilated despite balloon dilation. Therefore, a fully covered self-expandable metal stent (12 mm×6 cm) was placed to achieve adequate tract dilation ( Fig. 2c ). Eight weeks later, adequate tract dilation for POCS insertion was achieved after metal stent removal, and stone fragmentation was successfully performed using POCS-guided electrohydraulic lithotripsy (EHL; Fig. 3a and b ), resulting in complete stone clearance without any adverse events.

**Fig. 3:**
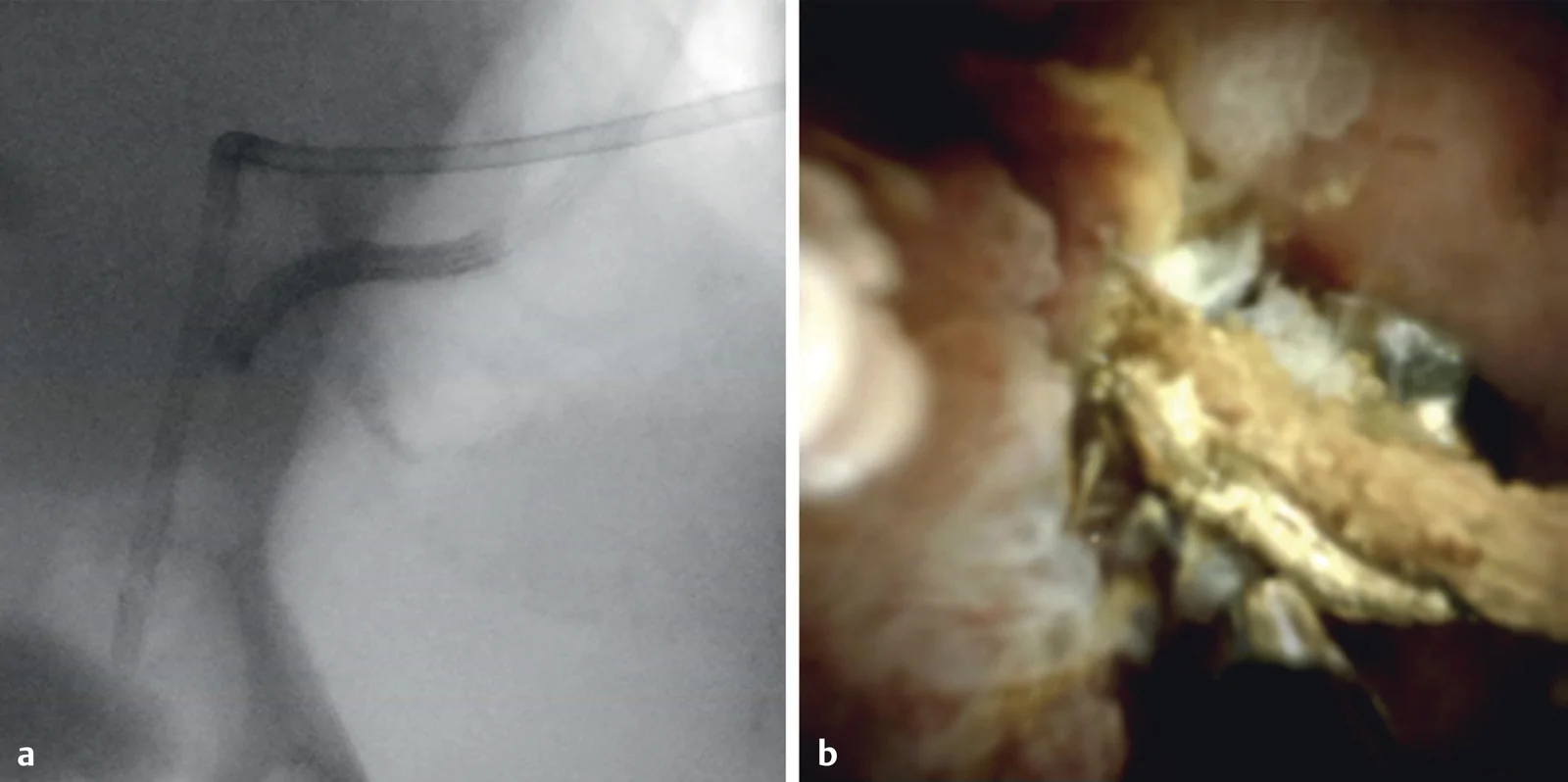
Cholangioscopy-guided electrohydraulic lithotripsy via the B1 EUS-HGS route. ( **a** ) A fluoroscopic image showing the insertion of the cholangioscope. ( **b** ) An endoscopic image demonstrating stone fragmentation.

EUS-HGS is recognized as an alternative treatment for patients with surgically altered anatomy in whom BE-ERCP is challenging. 1​ Typically, the B2/3 bile duct is selected as the target during EUS-HGS. 2​ Selective puncture of the anatomically challenging B1 bile duct enabled the complete removal of a large intrahepatic stone using POCS-guided EHL via a B1 EUS-HGS route, representing the first such reported case. This approach may represent a novel therapeutic option for difficult intrahepatic bile duct stones.

Endoscopy_UCTN_Code_TTT_1AS_2AD

## References

[1] Iwashita T Nakai Y Hara K Endoscopic ultrasound-guided antegrade treatment of bile duct stone in patients with surgically altered anatomy: A multicenter retrospective cohort study J Hepatobiliary Pancreat Sci 2016 23 227 233 26849099 10.1002/jhbp.329

[2] Ogura T Higuchi K Endoscopic ultrasound-guided hepaticogastrostomy: technical review and tips to prevent adverse events Gut Liver 2021 15 196 205 32694240 10.5009/gnl20096 PMC7960972

